# Body and Tail Coordination in the Bluespot Salamander (*Ambystoma laterale*) During Limb Regeneration

**DOI:** 10.3389/frobt.2021.629713

**Published:** 2021-05-28

**Authors:** Cassandra M. Donatelli, Keegan Lutek, Keshav Gupta, Emily M. Standen

**Affiliations:** Department of Biology, University of Ottawa, Ottawa, ON, Canada

**Keywords:** coordination, gait transition, limb loss, locomotion, embodiment, bio-inspired robotics, robotics-inspired biology

## Abstract

Animals are incredibly good at adapting to changes in their environment, a trait envied by most roboticists. Many animals use different gaits to seamlessly transition between land and water and move through non-uniform terrains. In addition to adjusting to changes in their environment, animals can adjust their locomotion to deal with missing or regenerating limbs. Salamanders are an amphibious group of animals that can regenerate limbs, tails, and even parts of the spinal cord in some species. After the loss of a limb, the salamander successfully adjusts to constantly changing morphology as it regenerates the missing part. This quality is of particular interest to roboticists looking to design devices that can adapt to missing or malfunctioning components. While walking, an intact salamander uses its limbs, body, and tail to propel itself along the ground. Its body and tail are coordinated in a distinctive wave-like pattern. Understanding how their bending kinematics change as they regrow lost limbs would provide important information to roboticists designing amphibious machines meant to navigate through unpredictable and diverse terrain. We amputated both hindlimbs of blue-spotted salamanders (*Ambystoma laterale*) and measured their body and tail kinematics as the limbs regenerated. We quantified the change in the body wave over time and compared them to an amphibious fish species, *Polypterus senegalus*. We found that salamanders in the early stages of regeneration shift their kinematics, mostly around their pectoral girdle, where there is a local increase in undulation frequency. Amputated salamanders also show a reduced range of preferred walking speeds and an increase in the number of bending waves along the body. This work could assist roboticists working on terrestrial locomotion and water to land transitions.

## Introduction

In nature, animals must adapt to a wide variety of perturbations to effectively move through their environments. Successful navigation of perturbations is necessary for finding food, escaping predation, reproduction, and nearly every other biological function requiring movement, meaning that animals have evolved to be behaviorally plastic (Beddard, 1902; Gillis and Blob, 2001; Dingemanse and Wolf, 2013; Touchon et al., 2013; Vega and Ashley-Ross, 2020). Even with advances in computational modeling, control algorithms, and robotics, technology is unable to match the behavioral flexibility of an animal in nature (Kim et al., 2013). This often results in models displaying unrealistic kinematics and robots getting stuck or damaged. Developing a greater understanding of how animals overcome obstacles in nature could provide useful data for roboticists to design more robust machines.

Fewer perturbations are more severe than losing a limb, yet salamanders are able to survive through the loss and subsequent regeneration of limbs in nature (Arenas Gómez et al., 2017; Dwaraka and Voss, 2021; Joven et al., 2019). After limb loss, the animal must adapt its locomotion to this acute perturbation and then continue to modify their behavior over weeks and months as the limb grows back. In some cases, the lost appendage never fully recovers (Dwaraka and Voss, 2021), which must then result in a permanent change in behavior. Though the limb regeneration process has been studied extensively in the context of cellular signaling, development, and phylogenetics (Arenas Gómez et al., 2017; Dwaraka and Voss, 2021; Joven et al., 2019), the change in kinematics has not been well described. We therefore chose to study how the kinematics of the Blue-spotted Salamander (*Ambystoma laterale*) changes during the limb regeneration process.

Salamander kinematics have been well studied in a wide range of scenarios from forward and backwards walking to aquatic to terrestrial transitions (Ashley-Ross, 1994; Azizi and Horton, 2004; Cabelguen et al., 2010; Sheffield and Blob, 2011; Karakasiliotis et al., 2013). During walking, there are three motions used for forward propulsion: 1) girdle rotation (10–18%), 2) limb rotation (26–28%), and 3) limb retraction (56–62%) where the percentages describe the amount each motion contributes to forward movement (Karakasiliotis et al., 2013). Girdle rotation is of particular interest as it is a result of local lateral bending of the vertebral column. Modeling with robots has confirmed that axial bending (i.e., girdle rotation) indeed plays an important role in walking and found that higher coordination between the limbs and vertebral column results in an increase in stride length (Karakasiliotis and Ijspeert, 2009; Crespi et al., 2013).

In water, salamanders change their gait entirely, moving from a sprawled tetrapod gait with limbs moving in an alternating stepping pattern to an undulatory swimming gait with limbs tucked against the body (Frolich and Biewener, 1992). This swimming mode is more like that of a fish than other tetrapods, such as dogs, which tend to use their limbs as paddles without whole body undulation (Rivera et al., 2011). This pre-programmed tendency to switch to body undulation when limb frictional or loading forces disappear, suggests that, when limbs are removed a similar increase in body undulation may occur. In addition, one might expect that removing the hind limbs of a salamander might elicit a terrestrial “walking” gait similar to that of amphibious fish that use pectoral fins for support but lack substantial pelvic fins such as the Senegal Bichir (*Polypterus senegalus*). We include data from *Polypterus* walking and swimming in this study for comparison.

Central pattern generators (CPGs) are neural circuits that can produce a patterned output without top down control (Duysens and Van de Crommert, 1998). CPGs control rhythmic movements such as walking, running, and swimming in both vertebrates and invertebrates and, along with local sensory feedback, make these movements robust to perturbations such as uneven terrain (Pfluger and Burrows, 1978; Kanou et al., 2007; Tytell et al., 2010; Garcia-Saura, 2015; Bidaye et al., 2018; Yasui et al., 2019). Local CPGs can be associated with axial and appendicular motion and decerebration and spinal transection studies have shown that CPGs with sensory feedback can remain active, resulting in effective locomotor function in the absence of signals from the brain (Zareen et al., 2016).

Since salamanders are one of the earliest diverging terrestrial tetrapods (Gueldre, 1992), it is thought that these axial CPGs are similar to those found in fish and other swimming vertebrates and that limb control evolved on top of the existing spinal CPGs (Chevallier et al., 2008). Computational and physical models of salamander gait transitions suggest that sensory feedback from the limbs causes the change in gait from a standing wave used in walking to an undulatory wave used in swimming (Ijspeert et al., 2007; Chevallier et al., 2008). This could mean that, without limbs, the salamander would return to a more fish-like undulatory gait. There is evidence for this in the kinematics of *S. lacertina*, a salamander species that does not have hindlimbs at all. During aquatic walking, its front limbs step in an alternating stepping pattern similar to the way the front limbs move during a tetrapod gait. However, its body moves in a traveling wave like swimming, as opposed to a standing wave like walking (Azizi and Horton, 2004). Amputation of limbs could have differing effects on an animal’s ability to locomote depending on the level of neuro-connection between body and limbs.

The field of robotics and control could learn a lot from how animals control motion and adapt to extreme perturbations such as the loss of one or more limbs (Trimmer and Lin, 2014; Chattunyakit et al., 2019; Kano et al., 2019). This is especially important for robots deployed in the field for long term missions such as deep sea or space exploration (Koos et al., 2013). There are existing algorithms for investigating new gaits after damage to a limb, but these are computationally expensive, require precise knowledge of the damaged part, and may result in further damage to the robot (Koos et al., 2013). Other options include pre-programming of gaits for specific limb-loss (Mostafa et al., 2010; Kano et al., 2019) but this may fail if the robot is damaged in an unexpected way. If we develop a deeper understanding of how animals use a combination of top-down control (CPGs), sensory feedback, and morphology to overcome extreme perturbations, this could be incorporated into future robotic design.

In this work we aim to understand more about how vertebrates change their locomotor patterns and possible control schemes to deal with perturbations by: 1) describing the change in body kinematics as the salamander regrows its hindlimbs, 2) discussing the control mechanisms that could drive these kinematic changes and 3) comparing the changing kinematics to the Senegal Bichir (*Polypterus senegalus*), an amphibious fish species in order to place this change in an evolutionary context.

## Materials and Methods

### Animals

We collected adult, Blue-spotted Salamanders (*Ambystoma laterale*) during their spring migration in the summers of 2018 and 2019. Though we cannot control for the exact age of wild caught animals, we can be sure that all animals are adults (Canadian Herpetological Society, 2021). All salamanders were collected locally during spring thaw as they crossed roadways to reach breeding ponds (Ottawa, Canada; collection permit 1092653). They were housed at the University of Ottawa aquatic animal facility under Animal Care Protocol number BL-1926.

### Surgery

**FIGURE 1:**
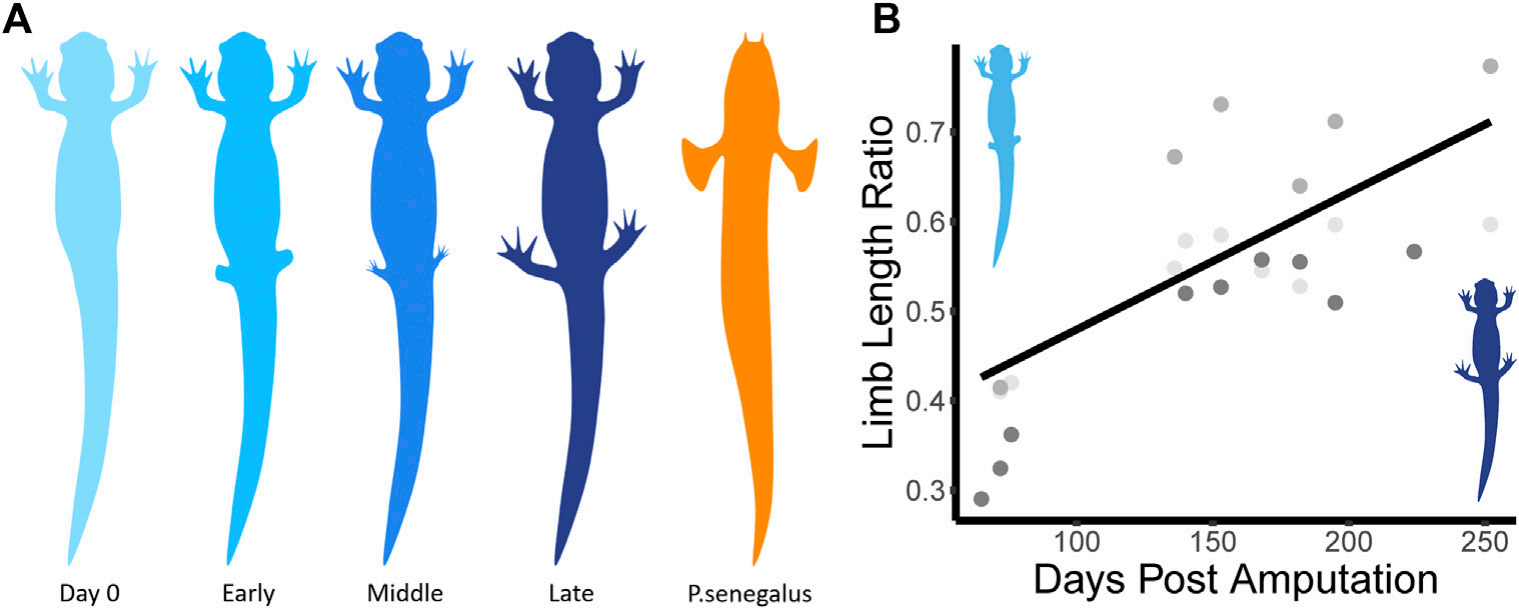
**(A)** Diagram of recovery process and other species used. There are three main stages to the limb recovery process. During the Early stage (light blue), limb buds have begun to grow back. During the Middle stage (medium blue), toes have re-grown. During the Late stage (dark blue), limb morphology is almost identical to pre-surgery, though, in this study, it never recovers its original length. We compared the kinematics of the salamander to *P. senegalus* (orange) swimming and walking trials. The color scheme will be consistent throughout the paper. **(B)** Shows the rate of limb regrowth (Limb Length Ratio, L_Current_/L_Original_) over time (Days Post Amputation). Different shades of grey represent different individuals.

Three individuals were chosen to undergo hindlimb removal surgery. We anesthetized them with a 0.1% ethyl 3-aminobenzoate methanesulfonate salt (MS222) for roughly 15 min or until they did not respond to stimuli. Once anesthetized, both hindlimbs were removed using a fresh scalpel blade (size 22) and pressure was applied to the wound to stop bleeding if required. Just after surgery, a lidocaine solution was infiltrated at the incision site and an intercoelomic injection of Buprenorphine (50 mg/kg) was administered for pain control. After surgery (Figure 1, Day 0), animals were allowed to recover for 1 month before trials. After 1 month, the wounds from surgery were healed and we could see that the regeneration process had begun (Figure 1, Early).

### Walking Trials

**FIGURE 2:**
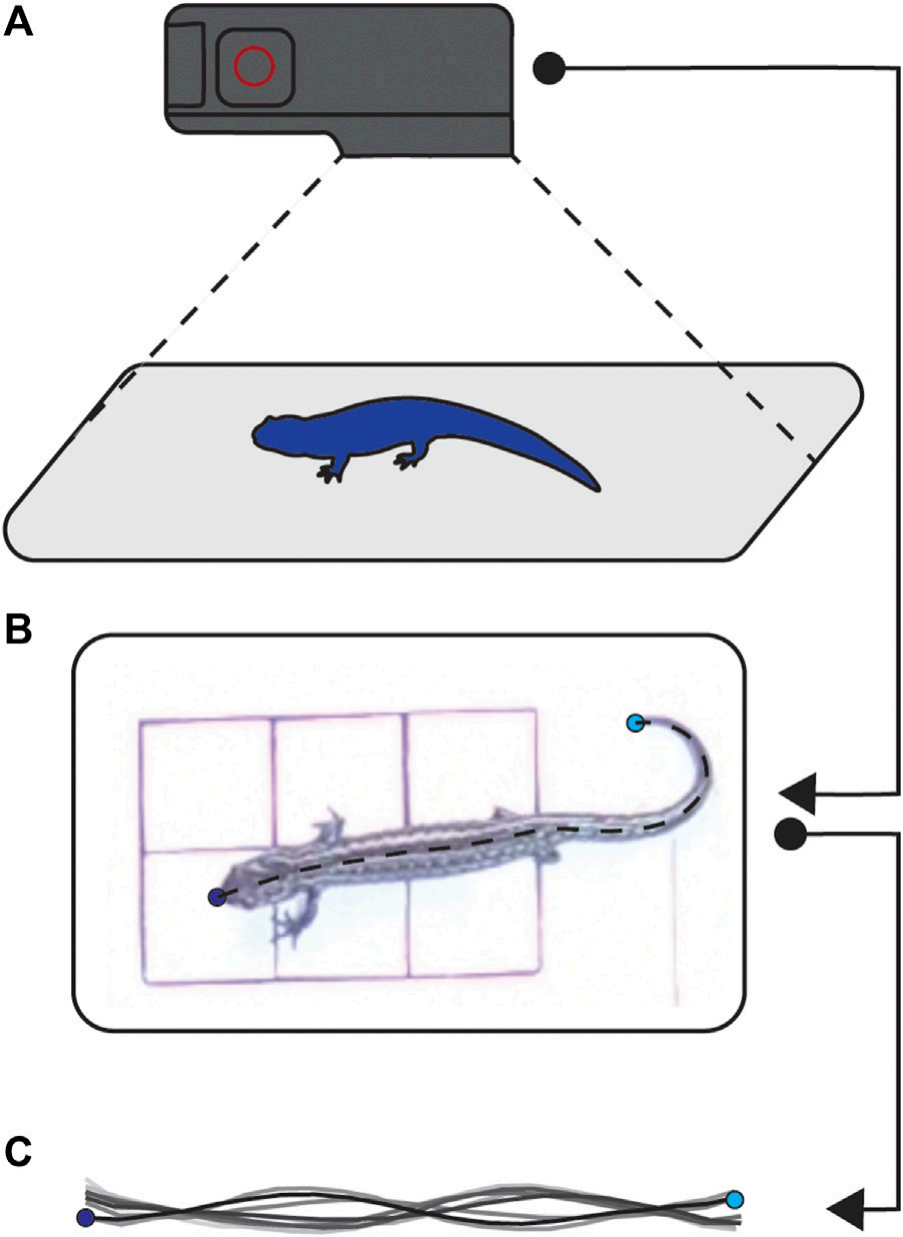
Filming setup. We used a GoPro to film salamanders from a top down view **(A)**. The resulting videos **(B)** were processed using DLTdv8 (Hedrick, 2008; Jackson et al., 2016) to track the nose and tail, and custom Matlab (v2020b, Mathworks, Needham, MA, United States) software in to produce midlines **(C)**.

We recorded bouts of walking for each salamander at 1- and 2-week intervals until their limbs were almost fully regenerated. When recording animal locomotion, we first transferred them in their home tanks from the housing facility to the filming room. Once in the filming room, we recorded salamanders walking, from above using a GoPro Hero 4 (GoPro Inc., San Mateo, CA, United States) at 120 frames per second resulting in a top-down view of walking bouts (Figures 2A,B). We filmed either until the salamander was no longer interested in walking or until we had five good runs. We considered a good run to have six full steps, and we excluded any trials with fewer steps post-filming.

### Kinematic Analysis

**FIGURE 3:**
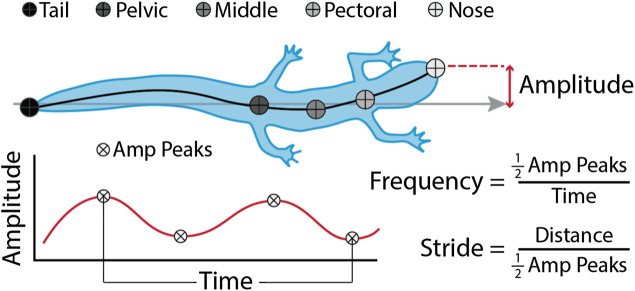
Kinematic variables. Amplitude (BL) and frequency (Hz) were measured at 21 evenly spaced points along the body (1 being the nose, 21 being the tail). Amp Peaks are the number of peaks in the amplitude wave, Time (s) is the length of time between the first and last peak, and Distance (BL) is the distance the nose of the salamander travels between the first and last peak. Statistics were done on the whole dataset, but some figures show only anatomical points of interest: nose, pectoral girdle, middle (halfway between the pectoral and pelvic girdles), pelvic girdle, and tip of tail. Stride length (BL) measurements were based on movement of the tail.

We measured midline kinematics of the salamanders walking using DLTdv8 (Hedrick, 2008) and our own custom software written in Matlab (v2020b, Mathworks, Needham, MA, United States). We first semi-automatically tracked the nose and tail using DLTdv8. Once those points were digitized, we used our own software to automatically trace the midlines. Our software requests user input to threshold the videos, then it converts them to binary, and uses the location of the nose and tail digitized in DLTdv8 to locate the animal and trace the midline. The midline tracing primarily uses the *bwskel* function from the Image Processing toolbox, which extracts the centerline and branches of binary objects. Once skeletonized, we use the nose and tail points to find the endpoints of the branches, choose the shortest path between the two, and smooth the resulting midline. Once we extracted the midlines, we used another Matlab script to measure body bending amplitudes (BL), and frequencies (Hz) of 21 evenly spaced points along the body (Figure 3). We also measured speed (BL/s), body waves (waves/s), and stride length (BL). Body waves is presented in waves/s to normalize for swimming speed. Stride length is defined as the distance the animal traveled during one tail beat cycle which allowed us to compare across conditions and between species. *Polypterus* walking and swimming data from a previous experiment were processed through the same code and used to compare with the salamander walking.

In addition to these kinematic variables, we also measured the change in limb length over time. Values are reported as “limb length ratio” and represent the current length of the limb (L_current_) as percentage of the original limb length (L_original_).

### Statistics

Kinematics data were imported into R (version 3.6.1) for statistics. We used the nlme and car packages to create linear mixed effect models. We chose to use linear mixed effects models since these models offer more flexibility when dealing with unequal sample sizes than, for example, a standard ANOVA. This flexibility allows us to use all data from each individual, rather than being forced to compute averages in order to have the same number of data points per sample. We created four mixed effect models. Our dependent variables were frequency, bending amplitude, speed, and stride length. All models included limb length ratio as a fixed effect. Our frequency, amplitude, and stride length models also included speed as a fixed effect. For all these models we included a random slope, modeled as days post amputation nested inside individual. These variables were chosen as random effects since each individual was recorded at similar time intervals and could be prone to random fluctuations in behavior on a day-to-day basis simply due to the variable nature of animal behavior. Individual must also be included as a random effect because of the repeated measures structure within our dataset. We also performed pairwise *t*-tests to determine differences between regeneration phases.

## Results

### Salamander Walking

Our first set of models looked at changes in kinematics as the limbs grew back. Here, limb length was included as a continuous variable (Table 1). Limb length ratio (L_Current_/L_Original_), walking speed (BL/s), and body position (%BL) had significant effects on body wave frequency (Hz) (*p* < 0.001 for all comparisons). Limb length ratio was a significant predictor of amplitude (BL) (*p* < 0.001) but position and speed were not (*p* = 0.703, *p* = 0.082, respectively). Limb length ratio was also a significant predictor of both walking speed (*p* < 0.001) and stride length (BL) (*p* < 0.001). Speed was also significantly affected by stride length (*p* < 0.001).

**TABLE 1:** LMER results.

	Predictors				
**y∼**	Limb length ratio	Days post amputation	Position	Speed	Stride length
Frequency	***p* < 0.001**	*p* = 0.065	***p* < 0.001**	***p* < 0.001**	*n/a*
Amplitude	***p* < 0.001**	***p* = 0.002**	*p* = 0.703	*p* = 0.082	*n/a*
Speed	***p* < 0.001**	***p* < 0.001**	*n/a*	*n/a*	***p* < 0.001**
Stride length	***p* < 0.001**	***p* = 0.023**	*n/a*	*n/a*	*n/a*

Bold entries indicate values that are significant (ie *p* < 0.05)

Our paired *t*-tests looked at differences between regeneration stages (Figure 1A; Table 2). These tests showed that there is a significant difference in mean bending frequency along the body between the Early regeneration stage and all other stages (*p* < 0.01 for all three comparisons). There is also a difference in mean stride length between the pre-amputation trials and the Early and Middle stages (*p* < 0.001 for both) but no difference between the pre-amputation trials and the Late regeneration stage trials.

**TABLE 2:** Paired *t*-test results. *p*-value adjusted using a Bonferroni correction. SDs were pooled.

	Frequency			Stride length		
	Early	Middle	Late	Early	Middle	Late
Middle	***p* < 0.001**	—	—	*p* = 1.00	—	—
Late	***p* = 0.011**	*p* = 1.000	—	*p* = 0.184	*p* = 0.247	—
PreAmp	***p* = 0.001**	*p* = 0.515	*p* = 0.317	***p* < 0.001**	***p* < 0.001**	***p* = 0.004**

Bold entries indicate values that are significant (ie *p* < 0.05)

**FIGURE 4:**
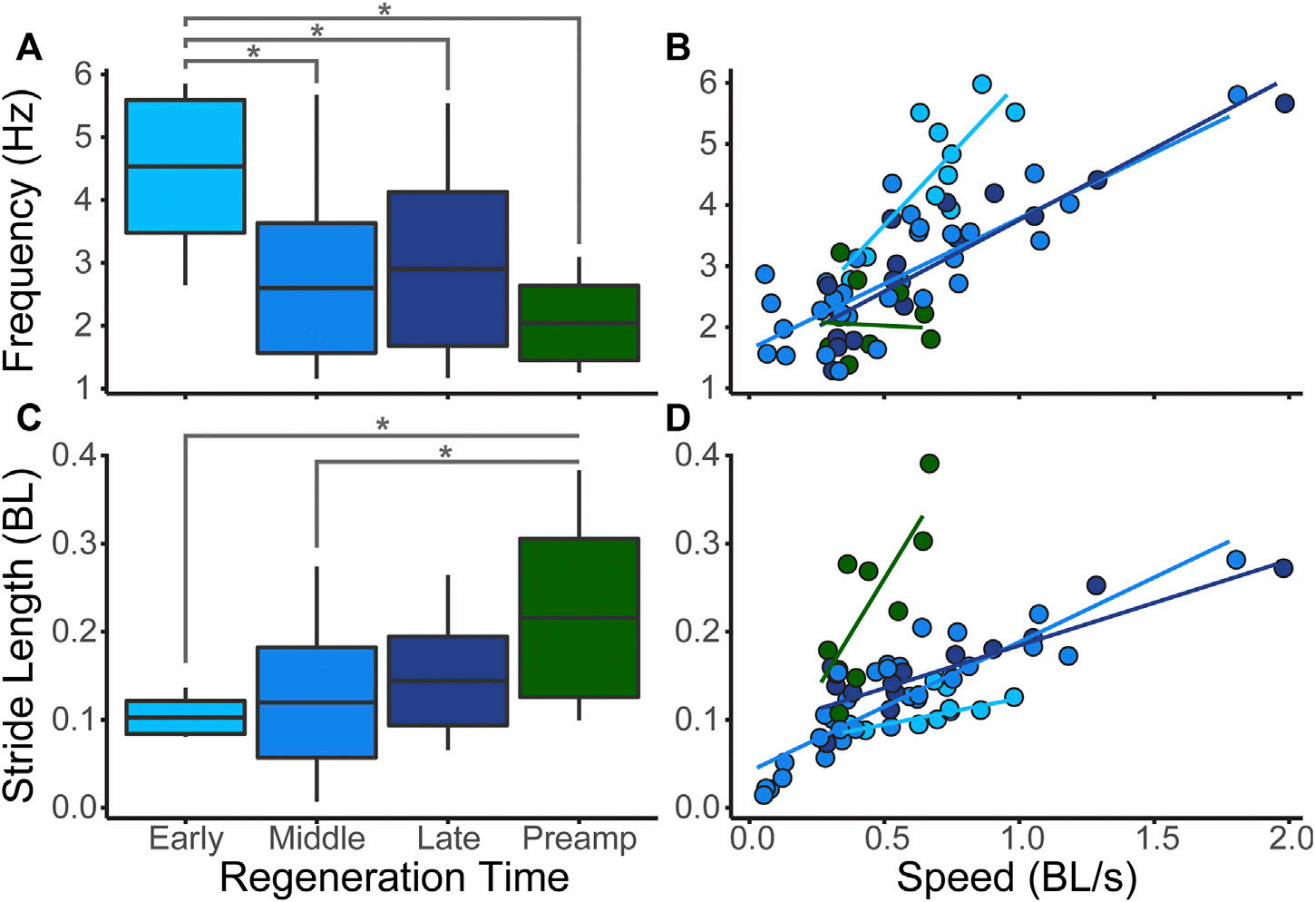
Salamander Kinematics. We measured walking speed (BL/s), bending frequency (Hz), and stride length (BL) over the course of limb regeneration. Significant differences between groups (*p* < 0.05) are represented by *. Boxplots show whole body medians, standard deviations, and minimum/maximum values. **(A)** Median body bending frequencies at the three regeneration time points plus the pre-amputation trials show that frequency is highest when the limbs are shortest. **(B)** Body frequency plotted against speed with fit lines show that frequency increases with walking speed. **(C)** Median stride length showing that stride length increases as limb length increases. **(D)** Stride length plotted against speed showing that stride length increases as speed increases.

In the early stages of regeneration, salamanders walk using a higher undulation frequency than salamanders in later stages (Figure 4A). Stride length increases steadily throughout the regeneration process (Figure 4C). As expected, as walking speed increases body undulation frequency and stride length also increase (Figures 4B,D).

### Comparison with another species

**FIGURE 5:**
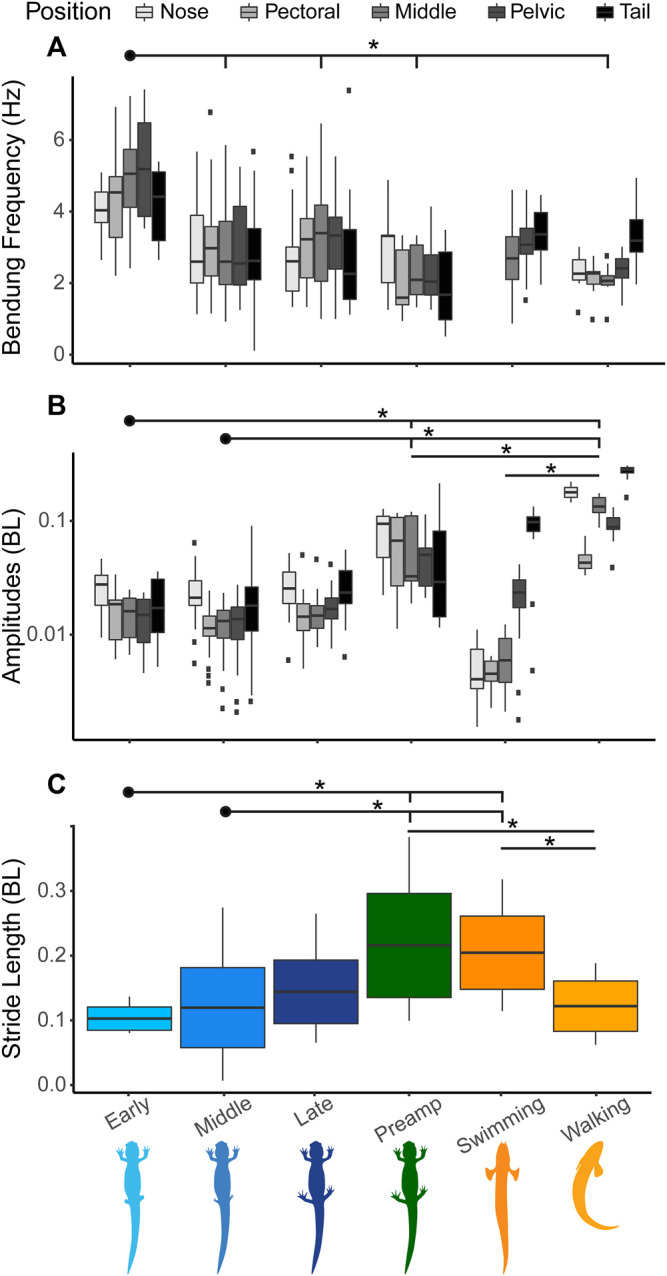
Salamander kinematics during recovery compared with fish kinematics. Kinematic patterns change as the salamander regenerates its legs. Significant differences between means (*p* < 0.05) noted by *. **(A)** Bending frequency (Hz) at five specific body points indicated by different shades of grey. From lighter to darker the shades indicate nose, pectoral girdle, middle, pelvic girdle, and tail. In our frequency plot (A.), we did not include the nose and pectoral girdle points for *Polypterus* swimming, as the low amplitude and flapping pectoral fins lead to noise in the frequency calculations. **(B)** Bending amplitude (BL) at five body points shown on a log scale. General trends show that portion(s) of the body used for propulsion have the lowest frequencies and highest amplitudes. **(C)** Stride length (BL) of salamanders at different time points compared with *P. senegalus* swimming and walking. The salamander data here is the same date presented in Figure 4C.

**FIGURE 6:**
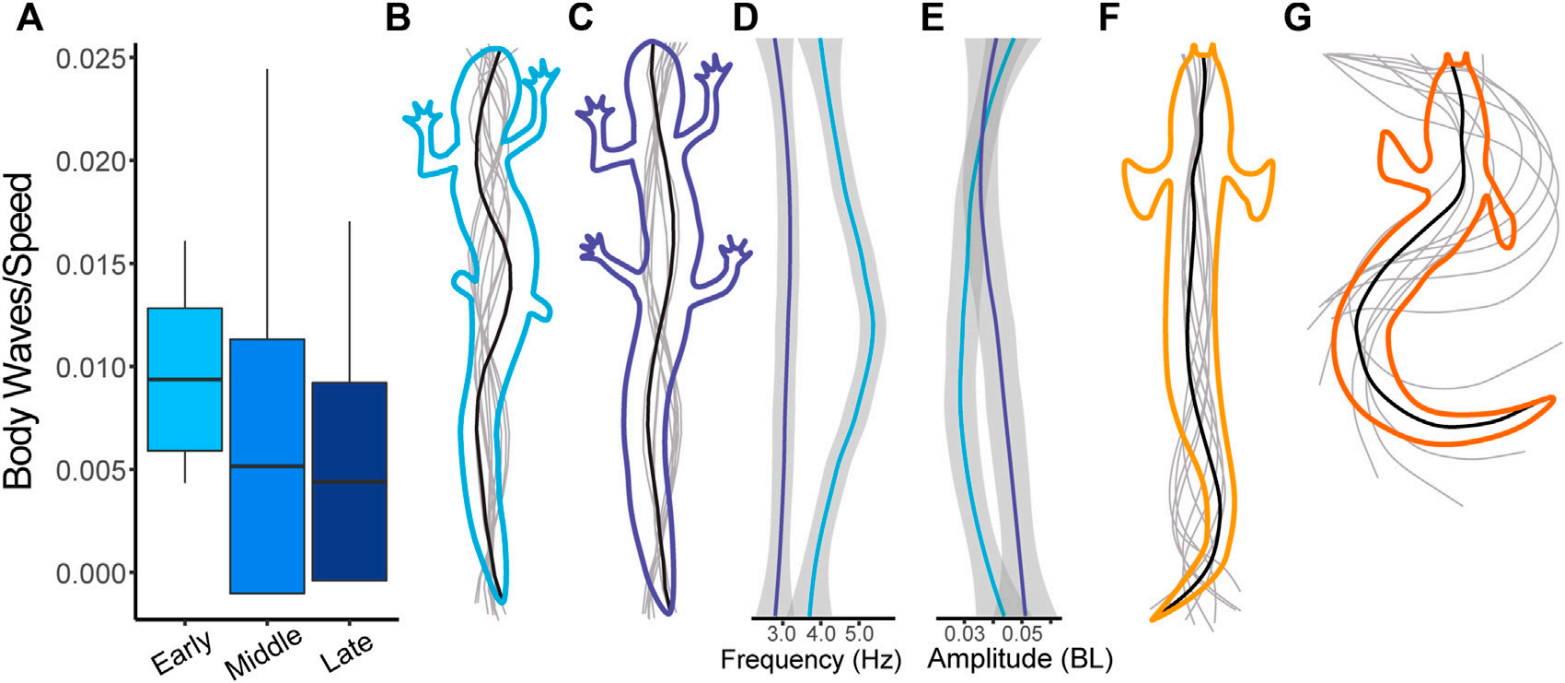
Change in kinematics between salamanders in the early and late stages of regeneration (blue) compared with *Polypterus* kinematics (orange). **(A)** Number of waves present on the body of the salamander scaled by walking speed). **(B)** Representative midline trace of a salamander walking in the early stages of regeneration. **(C)** Representative midline trace of a salamander walking in the late stages of regeneration. **(D)** Frequency (Hz) along the body of a salamander walking during early and late stages. **(E)** Amplitude (BL) along the body of a salamander walking during early and late stages. For D and E the top of the plot corresponds to the nose and the bottom to the tail. **(F)** Representative midline trace of a *Polypterus* swimming. **(G)** Representative midline trace of a *Polypterus* walking.

During the early stages of salamander limb regeneration, there is a change in the way their bodies move. For salamanders in the early stages of recovery, the frequency of the pelvic girdle is increased compared to other parts of the body (Figure 5A). As they recover, the frequencies of the pelvic and pectoral girdles begin to match more closely. For amplitudes, the pattern of salamander walking is consistent throughout recovery, with the highest amplitudes at the head and tail, though the nodes about which the amplitudes oscillate, change (Figures 5B, 6).

Overall, stride length increases as limbs regenerate with a significant difference between the Early and Middle stages of regeneration and the Pre-Amputation trials. Although we predicted that salamanders walking without hind limbs would resemble walking *Polypterus*, we find the opposite. With their limbs removed, the body kinematics of salamander walking is more like *Polypterus* or salamander swimming in that the number of waves along the body is increased. As the limbs regenerate, the kinematics shift and more closely resembles intact salamander walking prior to limb amputation.

## Discussion

### Salamander Kinematics Change During Regeneration

The most notable change in kinematics after hindlimb amputation in salamanders, is a shift in apparent undulation frequency of the pelvic girdle, the region where the hindlimbs articulate with the vertebra column. During the early stages of regeneration, the pelvic girdle sways back and forth at a frequency of 5 Hz while the rest of the body is swaying closer to 4 Hz (Figure 5A). Two potential explanations exist for this increase in frequency. First, without the sensory feedback associated with limb to ground contact, it is possible that *pelvic girdle CPG modulation changes.* Second, the increase in pelvic frequency may be due to a simple *mechanical constraint principle*.

If pelvic girdle CPG modulation is responsible for our observed change in kinematics, a change in local sensory feedback could be acting in two ways. If local force feedback from limb to ground contact inhibits axial (vertebral) CPGs in intact animals, the top-down (from the brain) signal that drives body oscillation would be uninhibited at the pelvis in amputated individuals, resulting in an increase in the speed of rotation of the hip. In contrast, if an absent or reduced sensory signal during regeneration is perceived as a misstep, the top-down signal could be “actively” increased, impacting local CPGs in the limbs and resulting in an increase in the speed of rotation of the pelvic girdle and local bending of the spine. Indeed, we observed that salamanders will occasionally take two steps with their reduced hindlimbs during a single front limb step, suggesting a perception of misstep may be the case. One could implant EMGs in the musculature along the body to investigate the change in CPG rhythm. If pelvic girdle CPG modulation changes are responsible for the increase in frequency, we would expect a change in intensity of the muscle signal at the pelvic girdle and/or an interruption in the standing wave of body muscle activation traveling from head to tail when the hindlimbs fail to contact the ground. If there is no change in axial muscle activation, the change in frequency must be due to mechanics.

One could also do an electrophysiology prep rather than use EMGs to measure the activation patterns of motor neurons directly. This type of work has been done in cockroaches and stick-insects using both *in-vitro*, fictive walking preps as well as semi-intact preps (Borgmann et al., 2009; Fuchs et al., 2011). The downsides of experiments like this are 1) for an *in-vitro* experiment, one would have to eliminate the mechanical perturbation of a limb hitting the ground. As a result, it would be hard to eliminate mechanical constraints from one’s conclusions. 2) The semi-intact prep procedure in vertebrates is incredibly invasive. There are ethical concerns regarding this type of procedure with vertebrates and the number of individuals one would need to use may be too high.

If the mechanical constraint principle is causing changing kinematics, we would expect the rhythm of the axial CPG to remain constant post amputation, which we could infer from axial EMGs. However, the mechanical constraint of the limb contacting the ground would be absent, allowing for more bending waves to be present along the body and an increase in rotation frequency at the girdle. Because there is no increase in the frequency of body sections not associated with lost limbs, mechanical constraint alone seems an unlikely explanation for the changes we see. It does not rule out the possibility that independent limb and axial CPGs at the girdles share in excitatory coupling, independent of sensory feedback, which, when free of mechanical constraint, increases the axial CPG oscillation signal locally at the limb (Delvolvé et al., 1997). This type of coupling has been shown in cockroaches, where an excitatory stimulation to one leg motor neuron results in coordinated activity in the ganglia of neighboring limbs (Fuchs et al., 2011). Other work on stick insects showed that front leg movement alone could activate descending pathways and coordinate the movement of the other limbs (Borgmann et al., 2009).

Interaction of limb and axial CPGs has been used to explain coordination between fore and hind limbs (Delvolvé et al., 1997). In this case, limb CPGs oscillate between increasing and decreasing the excitability of axial CPGs, resulting in the formation of coordinated limb motion and a standing body wave during walking. During the middle and late stages of regeneration, when limbs are regaining contact with the ground, the frequencies of both the pectoral and pelvic girdles sync at 3.5 Hz (Figure 5). Because girdles become phase locked regardless of the size of the regenerating limb, this data suggests a threshold control mechanism where sensory feedback from the limb is playing a role in helping to coordinate limb and body oscillation frequencies independent from the strength of the mechanical constraint on the system.

**FIGURE 7:**
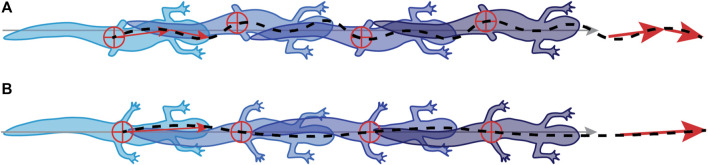
Salamanders use a different gait in early vs. late stages of limb regeneration. **(A)** During early stages of regeneration, salamanders shift their pelvic girdles from side to side at a higher frequency than the rest of the body, resulting in a gait that looks like a hybrid between a standing and traveling wave. **(B)** During later stages, they shift to a more typical standing wave.

Although sensory feedback may have an essential role in coordinating the walking cycle, mechanical constraint contributes to overall animal performance. During early stages of regeneration, the range of walking speeds is much lower than in the later stages (Figure 4D). Salamanders with no or very short hindlimbs have reduced stride lengths and a limited ability to raise their posterior trunk off the substrate. A higher speed may be necessary to overcome frictional forces and move at all, while shorter stride length limits distance per step cycle; both of these mechanically reduce the range of speeds that can be attained with reduced hind limbs (Figures 4B,D). In addition, the mechanical consequence of over rotation of the pelvic girdle, shifts the center of mass causing the head of the salamander to reflexively swing in the opposite direction correcting the overall path of the center of mass (Figure 7A). The result is that animals in the early stages of regeneration have a less optimal forward motion as the mass of the animal shifts more from side to side compared with later stage animals.

### Salamander Kinematics Resemble a Swimming Fish When Limbs Are Removed

Salamander swimming, like most undulatory fish swimming, is characterized by a traveling wave passing from head to tail, while walking is a standing wave with nodes at the pelvic and pectoral girdles. The amphibious fish, *Polypterus*, uses substantial pectoral fins to weight-bear when walking but lacks hind fin support. Instead, it uses an exaggerated full body and tail oscillation to push off the substrate (Figure 5B). We predicted, based on their similar body morphologies, that a salamander with its hind limbs removed might move like an amphibious walking fish, such as *Polypterus*. Contrary to this prediction, our data shows that, after leg amputation, salamander walking kinematics more closely resembles *Polypterus* swimming kinematics with an increase in the number of waves along the body (Figure 6). As limbs are more fully regenerated, there is a reduction in waves along the body and the gait more closely resembles the familiar standing wave associated with intact salamander walking as well as *Polypterus* walking. This may be the result of a universal control principle driving both salamander and *Polypterus* locomotion.

### Complexity in the Swimming to Walking Model and What It Means for Overall Vertebrate Motor Control

The CPG that controls undulatory swimming in the salamander is similar to that found in more early derived animals, such as the lamprey, suggesting that the activation pattern is constrained evolutionarily (Chevallier et al., 2008; Tytell et al., 2010). Even animals that predominantly use their fins for swimming, like *Polypterus* (Standen et al., 2014) often have accompanying, subtle asynchronous body and tail undulations suggesting that the basal swimming CPG is active in the background. When *Polypterus* swim faster, they tuck their fins against the body, and the more basal undulatory CPG appears to take over.

If the neural control scheme is similar between salamanders and walking fishes, models that explain the transition from swimming to walking in salamander (Ijspeert et al., 2007) could also explain the speed transition in swimming fish. When salamanders transition from walking to swimming, they tuck their limbs against their bodies and increase undulation frequency and amplitude just like a *Polypterus* increasing swim speed. The transition in *Polypterus* from swimming to walking, however, adds some complexity to the system because body oscillations increase and fin oscillation switches from synchronous oscillation to contralateral “stepping” (Standen et al., 2014; Standen et al., 2016). Similarly, salamanders switch from “synchronous” (inactive) limbs during swimming to contralateral stepping. If the underlying axial CPGs are always active, in both animals, mechanical constraints such as changes in friction and increased force regimes could be responsible for the differences in kinematics between slow swimming and walking.

Mechanical constraint may also influence the axial waveform of both *Polypterus* and salamanders as they move from an aquatic to terrestrial environment, or from having four to two limbs. In a salamander, limbs are used to lift the body away from ground frictional forces, focusing force constraints at the girdles, and driving the standing wave gait. In *Polypterus*, pectoral fins have limited ability to lift the body, thus frictional forces are experienced strongly by both fins and body, causing “stepping” in the pectoral fins and an increased axial oscillation in the tail. Even if the base signal from the brain to the axial CPGs remains a traveling wave, mechanical constraints at the pectoral and pelvic girdles in a salamander, and at the pectoral girdle and the tail in the fish, could constrain the traveling wave and cause a shift to a more standing form. In the salamander this becomes a true standing wave, while in the *Polypterus*, the posterior force concentration is more spread out and closer to the tail, resulting in a hybrid body wave that has both standing and traveling wave components. When the salamander lacks hind limbs, it too has a less concentrated posterior constraint. The elevation ability of the forelimbs reduces the impact of this change and the result is an increase in body waves that resemble the waves seen along the body during *Polypterus* swimming rather than walking. Interestingly, underwater walking in *S. lacertina*, a salamander that lacks hind legs, also shows a traveling axial body wave that accompanies fore-limb stepping (Azizi and Horton, 2004). All of this together could mean that the axial CPG is always active as the base controller for locomotion in vertebrates. The changes we see are only a result of changes in loading regimes which cause spikes in sensory feedback from various appendages and body parts.

### Insights for the Control of Limbed Robots

In nature, salamanders deal with perturbations, including limb loss, quickly and seamlessly enough to survive. Currently, there are few robots that can deal with such an extreme perturbation as the loss of two out of four limbs without pre-programmed or computationally expensive control regimes. When a salamander loses its limbs, the entire gait changes from a standing wave in intact salamanders, to a hybrid traveling-standing wave in amputated animals. The addition of these undulations at the nose and pectoral girdle seems to keep the center of mass of the animal moving forward (Figure 7). So, rather than focus on exact limb placement, the animal is prioritizing overall forward movement.

Limbed robots could use the same strategy to deal with damaged or lost limbs. Rather than pre-programming exact leg placements, there could be a greater focus on center of mass movement. Some robots, such as Salamander Robotica (Crespi et al., 2013) already have the ability to transition to a new gait when moving from land to water using sensory feedback and an increase in axial CPG frequency. A similar control scheme could be implemented when limbs are lost. Perhaps an inertial measurement unit (IMU) placed at the center of mass could drive rotations and undulations at key points, such as the nose, pectoral, and pelvic regions. Feedback from these points in conjunction with current advances in control algorithms (Santos and Matos, 2012; Koos et al., 2013; Kano et al., 2019) could allow the robot to tune a baseline axial CPG into a hybrid gait suitable for whatever force environment it’s in. Then, the robot could not only transition from aquatic to terrestrial locomotion but also deal with a range of perturbations from bumps in the road to loss of body parts. A robot with such a controller could be deployed to a much larger range of terrains, making it ideal for exploration of unknown environments.

## Data Availability

The raw data supporting the conclusions of this article will be made available by the authors, without undue reservation.
